# Neck Veins Don’t Lie: A Malignant Case of SVC Syndrome

**DOI:** 10.51894/001c.144473

**Published:** 2025-10-01

**Authors:** Alaa Ibrahim

**Affiliations:** 1 Department of Emergency Medicine Henry Ford Wyandotte

## 86

### INTRODUCTION

Superior vena cava (SVC) syndrome is a life-threatening condition caused by impaired venous return to the right atrium, most commonly due to malignancy. Malignancies account for approximately 60% of cases, with mediastinal lymphomas being a significant contributor. In non-Hodgkin lymphoma (NHL), SVC syndrome occurs in roughly 4% of cases, though aggressive subtypes, such as primary mediastinal large B-cell lymphoma, have an incidence as high as 60%. Given its potential for rapid deterioration, early recognition and timely intervention are essential to prevent complications such as cerebral edema and airway compromise. We present a case of SVC syndrome diagnosed in our community emergency department.

### CASE DESCRIPTION

A 32-year-old male presented with worsening dyspnea and orthopnea. He had recently been hospitalized for similar symptoms and diagnosed with a large mediastinal mass (8.6 × 12.2 × 17.1 cm) with pericardial extension, bilateral pleural effusions, and pericardial tamponade requiring drainage. Overwhelmed by his diagnosis, he left against medical advice before biopsy results were available.

On return to the emergency department, he was tachypneic and tachycardic, raising concern for recurrent tamponade or pulmonary embolism. Echocardiography showed a stable, small pericardial effusion without tamponade physiology. Upon re-evaluation patient developed facial plethora and distended neck veins, prompting concern for SVC syndrome. CT confirmed worsening SVC compression, right main pulmonary artery involvement, and bilateral pleural effusions. Chart review revealed a newly diagnosed high-grade B-cell lymphoma.

He was transferred to a tertiary center, initiated on R-CHOP chemotherapy with dexamethasone, and monitored for tumor lysis syndrome. Thoracentesis relieved dyspnea, and he was discharged on Day 3 of chemotherapy Cycle 1 with outpatient follow-up plans.

### DISCUSSION/CONCLUSIONS

This case underscores the importance of maintaining a broad differential diagnosis and reassessing patients with evolving symptoms. Unlike thrombotic SVC obstruction, malignancy-associated SVC syndrome necessitates urgent oncologic therapy rather than anticoagulation or stenting alone. In aggressive lymphomas, early chemotherapy initiation can rapidly alleviate symptoms and improve prognosis. Recognizing SVC syndrome as an oncologic emergency facilitates timely, multidisciplinary management, ultimately optimizing patient outcomes.

